# Actigraphy in Human African Trypanosomiasis as a Tool for Objective Clinical Evaluation and Monitoring: A Pilot Study

**DOI:** 10.1371/journal.pntd.0001525

**Published:** 2012-02-14

**Authors:** Alfred K. Njamnshi, Paul F. Seke Etet, Stephen Perrig, Alphonse Acho, Julius Y. Funsah, Dieudonné Mumba, Jean-Jacques Muyembe, Krister Kristensson, Marina Bentivoglio

**Affiliations:** 1 Neurology Department, Central Hospital Yaoundé/Faculty of Medicine, University of Yaoundé I, Yaoundé, Cameroon; 2 Department of Neurological Sciences (DSNNMM), University of Verona, Verona, Italy; 3 Sleep Studies Laboratory, University Hospitals, Geneva, Switzerland; 4 Institut National de la Recherche Biomédicale, Kinshasa, Democratic Republic of Congo; 5 Department of Neuroscience, Karolinska Institutet, Stockholm, Sweden; Institute of Tropical Medicine, Belgium

## Abstract

**Background:**

Human African trypanosomiasis (HAT) or sleeping sickness leads to a complex neuropsychiatric syndrome with characteristic sleep alterations. Current division into a first, hemolymphatic stage and second, meningoencephalitic stage is primarily based on the detection of white blood cells and/or trypanosomes in the cerebrospinal fluid. The validity of this criterion is, however, debated, and novel laboratory biomarkers are under study. Objective clinical HAT evaluation and monitoring is therefore needed. Polysomnography has effectively documented sleep-wake disturbances during HAT, but could be difficult to apply as routine technology in field work. The non-invasive, cost-effective technique of actigraphy has been widely validated as a tool for the ambulatory evaluation of sleep disturbances. In this pilot study, actigraphy was applied to the clinical assessment of HAT patients.

**Methods/Principal Findings:**

Actigraphy was recorded in patients infected by *Trypanosoma brucei gambiense*, and age- and sex-matched control subjects. Simultaneous nocturnal polysomnography was also performed in the patients. Nine patients, including one child, were analyzed at admission and two of them also during specific treatment. Parameters, analyzed with user-friendly software, included sleep time evaluated from rest-activity signals, rest-activity rhythm waveform and characteristics. The findings showed sleep-wake alterations of various degrees of severity, which in some patients did not parallel white blood cell counts in the cerebrospinal fluid. Actigraphic recording also showed improvement of the analyzed parameters after treatment initiation. Nocturnal polysomnography showed alterations of sleep time closely corresponding to those derived from actigraphy.

**Conclusions/Significance:**

The data indicate that actigraphy can be an interesting tool for HAT evaluation, providing valuable clinical information through simple technology, well suited also for long-term follow-up. Actigraphy could therefore objectively contribute to the clinical assessment of HAT patients. This method could be incorporated into a clinical scoring system adapted to HAT to be used in the evaluation of novel treatments and laboratory biomarkers.

## Author Summary

The clinical picture of the parasitic disease human African trypanosomiasis (HAT, also called sleeping sickness) is dominated by sleep alterations. We here used actigraphy to evaluate patients affected by the Gambiense form of HAT. Actigraphy is based on the use of battery-run, wrist-worn devices similar to watches, widely used in middle-high income countries for ambulatory monitoring of sleep disturbances. This pilot study was motivated by the fact that the use of polysomnography, which is the gold standard technology for the evaluation of sleep disorders and has greatly contributed to the objective identification of signs of disease in HAT, faces tangible challenges in resource-limited countries where the disease is endemic. We here show that actigraphy provides objective data on the severity of sleep-wake disturbances that characterize HAT. This technique, which does not disturb the patient's routine activities and can be applied at home, could therefore represent an interesting, non-invasive tool for objective HAT clinical assessment and long-term monitoring under field conditions. The use of this method could provide an adjunct marker of HAT severity and for treatment follow-up, or be evaluated in combination with other disease biomarkers in body fluids that are currently under investigation in many laboratories.

## Introduction

Human African trypanosomiasis (HAT), commonly known as sleeping sickness, is caused by subspecies of the protozoan parasite *Trypanosoma brucei (T.b.)* and transmitted by tsetse fly bites in sub-Saharan Africa. This disease, which mainly affects rural populations, is one of the most neglected tropical diseases, and is fatal if left untreated [1]–[3]. There are two forms of the disease: the West and Central African form caused by *T.b. gambiense*, which represents the vast majority of cases, and the East African form caused by *T.b. rhodesiense.* The disease evolves in two stages: hemolymphatic (stage 1) and meningoencephalitic (stage 2), which require different treatments. The arsenical compound melarsoprol has been widely used for stage 2 disease, but this therapeutic approach has severe side-effects, including fatal complications [3]. The main criterion for disease staging relies principally on the detection of elevated white blood cell (WBC) number and/or trypanosomes in the cerebrospinal fluid (CSF) [4]. The validity of this criterion is, however, under debate [5]–[8], information on its correlation with clinical disease severity is limited, and new laboratory biomarkers for disease staging are currently under evaluation [7], [9]–[13]. Objective clinical methods, preferably non-invasive, are therefore needed not only for the examination of HAT patients but also for follow-up, correlation with stage biomarkers, evaluation of treatment results and assessment of clinical trials.

HAT leads to a constellation of neurological and psychiatric alterations, with characteristic sleep disturbances [2], [14]–[18]. In fact, in a prospective multinational study of a large cohort of HAT patients, sleeping disorder, subjectively reported by the patients mostly as insomnia, was found to be the leading clinical symptom of the disease [17]. Sleep alterations have been repeatedly proposed as main signs of central nervous system involvement in HAT [3], [15], [19], although their relationship with HAT staging is not yet fully established.

The technique of polysomnography (PSG) has documented in detail sleep disturbances in HAT [14]–[16], showing that the disease is characterized by disruption of the sleep-wake cycle during 24 h, with nocturnal insomnia and daytime sleepiness, and thus drawing attention also to disturbances of daily biological rhythms. The structure of sleep, and especially the sequence of the two types of sleep, namely rapid eye movement (REM) sleep which should be normally preceded by non-REM (NREM) sleep, is also frequently altered in HAT, with the occurrence of sleep-onset REM (SOREM) episodes [15] in which REM sleep is preceded by wakefulness. These disturbances are improved or reversed by treatment, indicating that they are disease-related signs [15], [20]. PSG has been proposed as a non-invasive technique also for the evaluation of children affected by HAT [19]. However, PSG recordings are cumbersome, relatively expensive and difficult to perform in resource-limited health centers, and may not, therefore, represent a routine procedure for HAT in the field. This has led us to the search for less expensive and more user-friendly non-invasive technologies to allow objective assessment of day/night disturbances during the disease. A method developed in the last years is actigraphy, based on movement recordings via battery-run activity sensors, the actigraphs, wrist-watch size devices mostly worn on the wrist. Collected data are then downloaded to a portable computer and analyzed to provide an estimate of alterations of rest-activity from which sleep-wake parameters are analyzed [21], [22].

Actigraphy has been developed and validated for human circadian rhythm disorders and sleep disturbances. Although actigraphy does not reach the level of information on sleep and wake parameters obtained by PSG, this technique provides a non-invasive, reliable tool for the ambulatory assessment of sleep disorders and the effects of treatment designed to improve sleep [21], [23]–[25]. Actigraphy is used in a variety of clinical conditions (see, for example, [26]–[28]), and has been increasingly used also in children [21], [29].

According to WHO guidelines, second stage HAT is defined by the detection in the CSF of >5 WBC/µl [4], but different cut-off criteria are used in some African countries where HAT is prevalent. In particular, a parameter of >5 up to ≤20 WBC/µl CSF, defining an intermediate stage of disease, is also used before second stage drug treatment is initiated [8], [17], [30], [31]. Since objective tests of HAT symptoms are urgently needed for clinical assessment of the patients and treatment follow-up under field conditions, we here undertook a pilot study based on actigraphic recordings of patients infected by *T.b. gambiense* in different stages of disease.

The investigation was conducted in two phases. First, actigraphy was performed in Cameroonian patients before and after the initiation of treatment. These findings have been previously reported in part in abstract form [32]. The study was then pursued, in the Democratic Republic of Congo (DRC), with actigraphic recordings of HAT patients at admission. Nocturnal PSG was recorded simultaneously to actigraphy. All the parameters derived from actigraphy have been analyzed with a user-friendly software which can be easily applied under field conditions. Altogether the findings point to actigraphy as a useful, non-invasive tool for an objective clinical assessment and monitoring of HAT.

## Materials and Methods

### Study sites and patient recruitment and clinical evaluation

Two patients (Y1 and Y2) were investigated and treated in the Neurology Department of the Central Hospital of Yaoundé, Cameroon (Table 1). This is one of the countries known to be endemic for HAT, classified among those with less than 100 new cases per year [6] with 5 foci of the disease [33], [34]. The patients were referred by the National Trypanosomiasis Control Programme of the Ministry of Public Health during a screening campaign for HAT after positive screening for antibodies against *T.b. gambiense* in the blood with the card agglutination test for trypanosomiasis (CATT). Both patients presented with histories of various complaints, including nocturnal insomnia and diurnal somnolence. CSF analysis was done according to standard methods at the Centre Pasteur Laboratory in Yaoundé. On the basis of the CSF finding of 6 WBC/µl, the patients were diagnosed as intermediate stage HAT (see above), and were treated with a 10-day course of pentamidine (4 mg/kg daily, administered through the deep intramuscular route). The drug was supplied free of charge through the World Health Organization system.

**Table 1:** Data on subjects investigated in the present study.

	Y1	Y2	K1	K2	K3	K4	K5	K6	K7
**Age (years)**	39	27	5	30	50	51	62	45	25
**Sex**	M	F	F	F	F	F	M	F	M
**Country of origin**	CMR	CMR	DRC	DRC	DRC	DRC	DRC	DRC	DRC
**CSF WBC count** *	6	6	3	5	6	6	27	935	1150
**Disease stage**	int.	int.	1	1	int.	int.	2	2	2

	CY1	CY2	CK1	CK2	CK3	CK4	CK5	CK6	CK7
**Age (years)**	42	27	5	30	43	50	62	47	27
**Sex**	M	F	F	F	F	F	M	F	M
**Country of origin**	CMR	CMR	DRC	DRC	DRC	DRC	DRC	DRC	DRC

*Trypanosoma brucei were not detected in any of the CSF samples.

Abbreviations: CMR: Cameroon; CSF: cerebrospinal fluid; DRC: Democratic Republic of Congo; int: intermediate; WBC: white blood cell.

Actigraphic recordings were performed at admission after the initiation of treatment. The patients kept a diary of daily activities throughout the investigation. Nocturnal PSG was recorded in patient Y2 simultaneously with actigraphy before and after treatment. Actigraphy of healthy, age- and sex-matched control subjects (CY1 and CY2) was recorded in parallel with the first actigraphic recordings of patients Y1 and Y2.

Seven patients (K1–K7; Table 1) were investigated in DRC, which is classified to be among the countries with more than 1000 HAT cases per year [6], [34] and with considerable underreporting [35]. All the patients had just come to observation through the National Sleeping Sickness Control Programme (NSSCP) of the Ministry of Health and had not yet received specific treatment. Also these patients were found to be infected by *T.b. gambiense* with CATT screening and disease stages were defined on the basis of CSF analysis as above (Table 1). Nocturnal PSG recordings were performed simultaneously with actigraphy, upon arrival, in the “Institut National de la Recherche Biomédicale” (INRB) in Kinshasa. The patients were then transferred to a hospital in Kinshasa (“Centre Hospitalier Roi Baudouin I” or “Centre Neuro-Psycho-Pathologique”, CNPP), in collaboration and conformity with the procedures of the NSSCP for initiation of treatment. Actigraphic recordings were also done, in the same environments, on age- and sex-matched control subjects (CK1–CK7), who were CATT-negative.

The patients had a thorough general and neurological examination. HIV serology was negative in all the patients and control subjects.

### Actigraphic and polysomnographic recordings

For actigraphy, octagonal BASIC Motionlogger® actigraphs (Ambulatory Monitoring, Inc., New York, NY, USA) were worn by the subjects on the non-dominant wrist. Throughout the recording period, the actigraphs were only removed when the subject was taking a shower to avoid damage by water to the device.

For PSG, electrodes were placed to record the electroencephalogram (EEG), electrooculogram, and electromyogram according to standard procedures (see also [15]). The standard 10/20 system was used to place EEG electrodes on the scalp of the subjects. A computerized system equipped with Neuron Spectrum PSG software (Neuronsoft®, Geneva, Switzerland) was used to filter, record, and store the tracings obtained.

In patient Y1, 24 h actigraphic recordings were done upon admission and at day 55 after the initiation of treatment. In patient Y2, 36 h actigraphic recordings were performed before treatment and 14 days after the initiation of treatment, and 12 h PSG recordings were performed simultaneously with actigraphic recording during the night. In patients K1–K7, 36 h actigraphic recordings were performed at presentation, together with 12 h PSG recordings during the night.

No difficulties were encountered during actigraphic recordings, whereas for PSG several challenges were faced at the Neurology Department of the Yaoundé Central Hospital as well as at INRB in Kinshasa, especially due to electrical power failures (which also caused non-recording or loss of PSG data for patients Y1 and K7).

### Ethics statement

The study was conducted according to the principles expressed in the Declaration of Helsinki. All patients recruited received written and verbal information explaining the purpose of the study and gave informed consent. Ethical consent forms were designed in English and French in Cameroon and in French in DRC, and were also translated into local languages during administration. For the participation of the 5 year-old patient and matched control child, consent was given by the parents. The protocols received approval and ethical clearance by the Cameroon National Ethics Committee, and authorization by the Ministry of Public Health of Cameroon, as well as by the National Ethics Committee of the Democratic Republic of Congo and the Ministry of Health National Sleeping Sickness Control Program. All patients were hospitalized and cared free of charge in the Neurology Department of the Central Hospital Yaoundé for the Cameroonian patients and in the “Centre Hospitalier Roi Baudouin I” or CNPP for the Congolese patients. All hospitalization charges were paid by the research project funds.

### Data analysis and statistical evaluation

**Figure 1:**
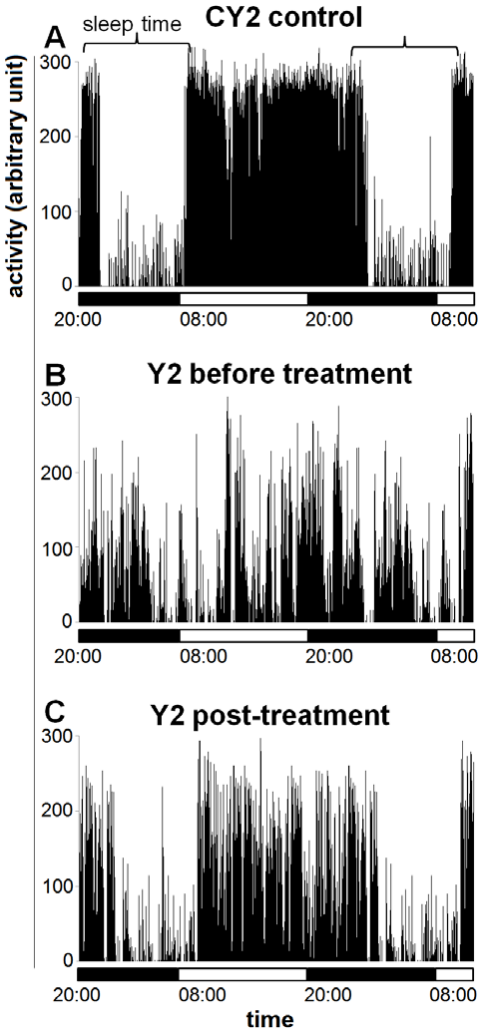
Actigrams of a patient and of a control subject. Actigrams are 36-h long, and correspond to tracings from the control subject CY2 (A) and patient Y2 before treatment (B) and at 14 days after initiation of treatment (C). The horizontal bars indicate the light and dark periods.

The raw data of activity over time (actigram) were displayed on a portable computer (Figs. 1 and S1). Analysis of the actigrams was performed with the Action 4 version 1, a user-friendly software supplied by the manufacturer of the actigraphs (Fig. S1). Following the software steps (Fig. S1), the algorithm of Sadeh [22], [36] was applied to the rest-activity (RA) signals to estimate the total sleep time during the night (the daily suggestive period of rest: from 10 PM to 7 AM), and the day (the daily suggestive period of activity: from 10 AM to 7 PM).

In addition, using the same software (and the steps illustrated in Fig. S1), raw activity values were analyzed to obtain information on the 24 h RA rhythm using the cosinor rhythmometry method (see supporting information S2). The Action 4 software, as well as software provided by other actigraph manufacturers allow to perform such analyses in a few rapid steps which can be easily learned without extensive training. Cosinor rhythmometry analyses provide an F-ratio, which reflects the degree of fragmentation of the RA signal: the more the tracing is fragmented, the lower is the associated F-ratio. Data were also obtained on the characteristics of the RA rhythm and, in particular, the rhythm-adjusted mean (MESOR), amplitude, and peak activity time or acrophase [37], [38] (see supporting information S2).

The PSG tracings were scored and analyzed using the Neuron Spectrum PSG software mentioned above, and the total sleep time during the night (from 10 PM to 7 AM) was determined.

Statistical evaluation was conducted on data derived from the recordings performed in DRC, for which groups of subjects were available. The sleep total time during day or night, respectively, was evaluated with the Student's unpaired *t* test in the adult patients K2–K7 *versus* the matched control group of adult subjects (CK2–CK7). P values lower than 0.05 were considered significant.

## Results

### HAT patients in Cameroon

On observation of 24 h actigrams of patient Y1 before treatment, bouts of high activity were evident during the night, indicating disturbed sleep. The actigram of patient Y2 showed more marked alterations, which consisted not only of bouts of high activity during the night but also of a fragmented activity during the day, frequently interrupted by episodes of rest indicative of episodes of diurnal somnolence (Fig. 1B), as compared to the control (Fig. 1A).

**Figure 2:**
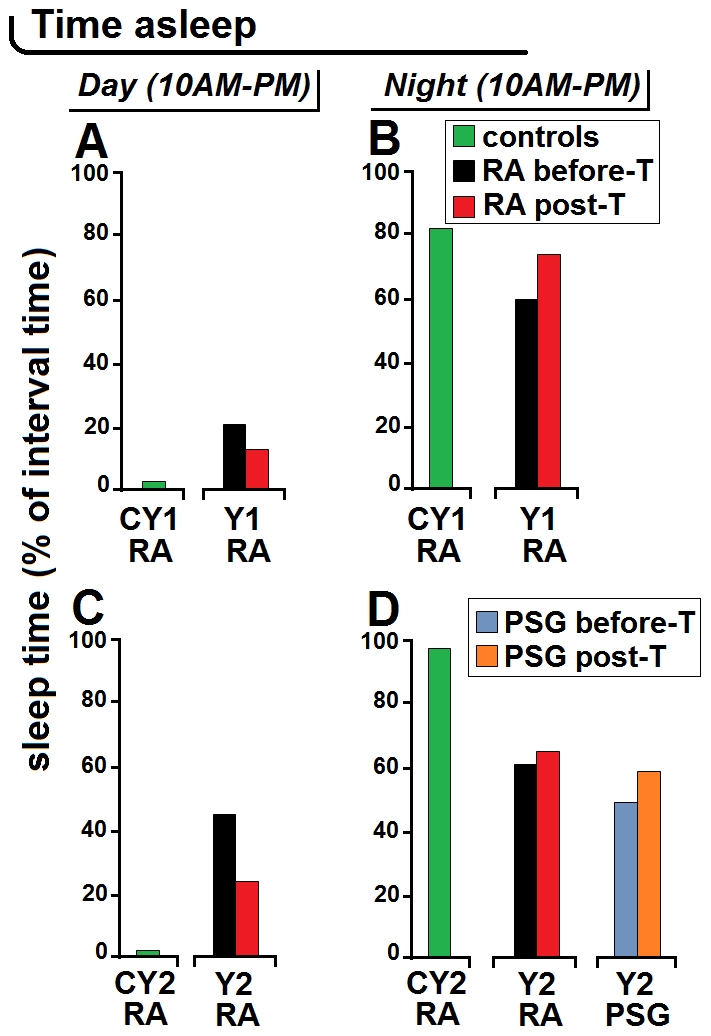
Total sleep time estimated from actigraphic recordings. The total sleep time was estimated from actigraphic recordings (RA, rest-activity) in control subjects CY1 and CY2, and in patients Y1 and Y2 before and after treatment (T) initiation and, in patient Y2, also with simultaneous nocturnal polysomnography (PSG). “Day” corresponds to the suggestive daily time of maximal activity and “night” to the suggestive daily interval of rest. A,B illustrate data obtained in patient Y1 and matched control subject; C,D illustrate data obtained in patient Y2 and matched control subject.

Quantitative analyses of the actigrams recorded in these patients before treatment showed that in patient Y1 the total time spent asleep corresponded to 20% of the day (*versus* 2% in the matched control subject) and 59% of the night (*versus* 81% in the control) (Fig. 2A,B). In patient Y2 the time spent asleep corresponded to 44% of the day and 60% of the night (*versus* 1.8% and 96.3%, respectively, in the matched control subject) (Fig. 2C,D).

The alterations revealed by the actigraphic recordings showed an improvement after the initiation of treatment. In patient Y1 the number and amplitude of bouts of activity during the night decreased with respect to the pre-treatment recording. Analysis of the actigram at 55 days after treatment showed that the total time spent asleep had decreased to 12% of the day and increased to 73% of the night (Fig. 2A,B). In patient Y2, the actigram pattern seemed to have improved especially during daytime at 14 days after treatment (Fig. 1C), as confirmed by the analyses which showed a marked decrease of the time spent asleep during the day (23%) and a slight increase of the sleep time at night (64%) (Fig. 2C,D).

**Figure 3:**
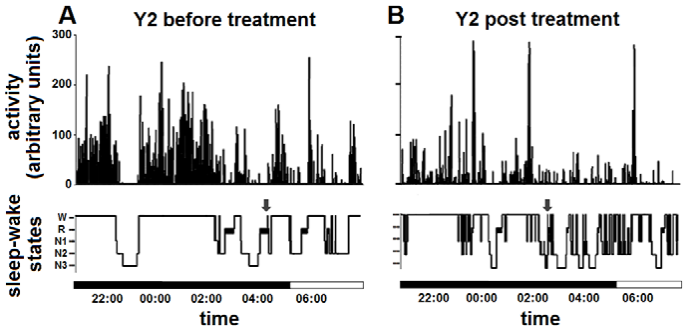
Comparison of nocturnal actigrams and hypnograms. The nocturnal actigrams (12-h long) (upper) and corresponding hypnograms (lower) of patient Y2 before (A) and after (B) treatment initiation are shown. Note in A the long bouts of activity corresponding to wakefulness (W) which therefore cause sleep disruption; note in B the improvement of these signs of disease (decrease of night activity with improvement of the hypnogram pattern) at 14 days after treatment initiation. Note also the close correspondence between actigrams and hypnograms, with the exception of sleep-onset REM sleep (SOREM) episodes (arrows) detected in the hypnogram only. The horizontal bars indicate the light and dark periods. Other abbreviations: R: rapid eye movement (REM) sleep, N1–N3: phases of non-REM sleep.

Nocturnal PSG recordings were also made in patient Y2, so that the actigram and concomitant hypnogram could be compared (Fig. 3). The analysis of sleep time from PSG indicated that before treatment patient Y2 spent asleep 47% of the night, which increased to 58% at two weeks after treatment (Fig. 2D). Such values indicated that in this patient actigraphy had overestimated the proportion of sleep during the night as compared to PSG, which, as it will be discussed further, is a possible limitation of this technique [22]. Nevertheless, both RA and PSG recordings showed a reduction of the patient's sleep time during the night with an improvement after treatment, though actigraphy underestimated the post-treatment improvement of this parameter.

The nocturnal actigrams and hypnograms showed a good correspondence in patient Y2, in particular revealing activity bursts and wakefulness episodes (Fig. 3). However, SOREM episodes observed in the hypnogram before and after treatment (Fig. 3) were not shown by the actigrams. This is due to the fact that, as also discussed further, algorithms used to estimate sleep from RA consider short intervals of activity and are therefore not suited for the detection of brief, single epoch events [22], such as SOREM events.

**Figure 4:**
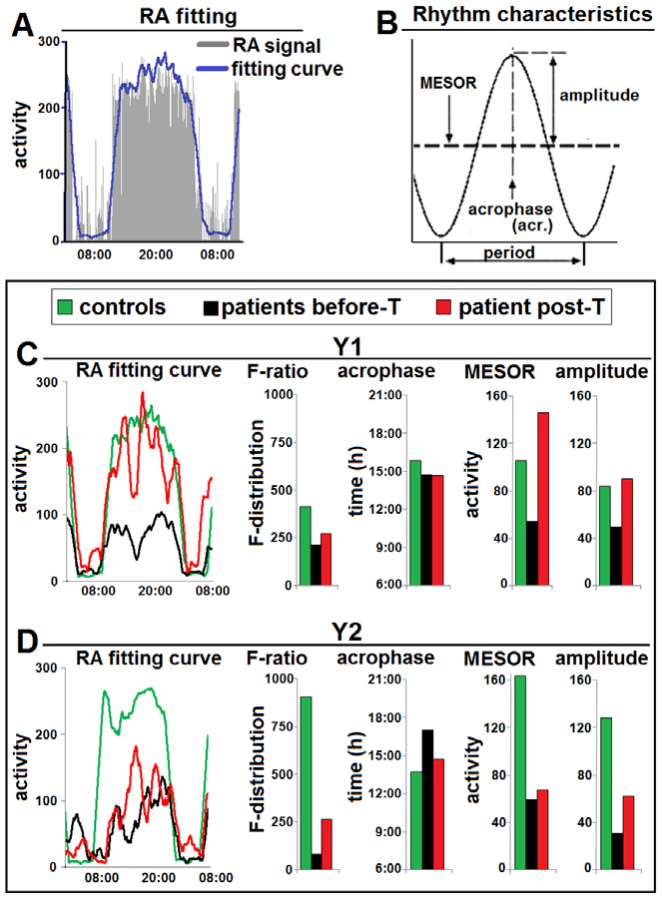
Characteristics of the rest-activity rhythm in patients Y1 and Y2. A. Illustration of a RA tracing fitting curve (obtained with the software provided by the manufacturer of the actigraphs: see Figure S1) in a healthy subject (CY1). Note that it gives at a glance the graphic demonstration of the daily waveform pattern. **B.** Illustration of the characteristics of a rhythm. These parameters are automatically computed using cosinor rhythmometry (see supplementary information S2). **C.** Parameters of patient Y1 before treatment (T) and at 55 days after treatment initiation, compared with control subject CY1. **D.** Parameters of patient Y2 before treatment and 14 days after treatment initiation, compared with control subject CY2. As explained in the text (see also supplementary information Text S1), the F-ratio value (value of associated F-test) provides an estimate of the characteristics of the rhythm shown by the actigram waveform: the more the RA tracing is disrupted the lower is the F-ratio value. Note in C and D the alterations of the waveform, and the post-treatment improvement of its pattern (which, however, is still altered), with corresponding increase of the F-ratio. Note also the alterations of rhythm characteristics (with decrease of MESOR and amplitude) which improve after treatment.

Analysis of the RA signal and rhythm characteristics (Fig. 4A,B) provided further objective demonstration of disease-related signs in these patients. The waveform of the activity data (fitting curve) allowed the visualization of the daily curve of the rhythm (Fig. 4A). This clearly showed an alteration in patients Y1 and Y2 at admission, which was less marked, especially in patient Y1, after treatment (Fig. 4C,D). The value of the F-ratio, which was decreased in both patients and especially in patient Y2, also showed a post-treatment increase, indicating clinical improvement (Fig. 4C,D). In particular, before treatment the F-ratio value was 228 in patient Y1 (446 in control subject CY1), and 87 in patient Y2 (970 in control subject CY2). During treatment this value increased to 293 in patient Y1 and 286 in patient Y2. Evaluation of the rhythm characteristics (acrophase, MESOR and amplitude) showed different degrees of alteration in the patients and partial recovery after treatment (Fig. 4C,D).

### HAT patients in DRC

As mentioned previously, 36 h actigraphy and simultaneous 12 h nocturnal PSG were performed in patients K1–K7, including one child (K1), who were analyzed at admission (Table 1).

**Figure 5:**
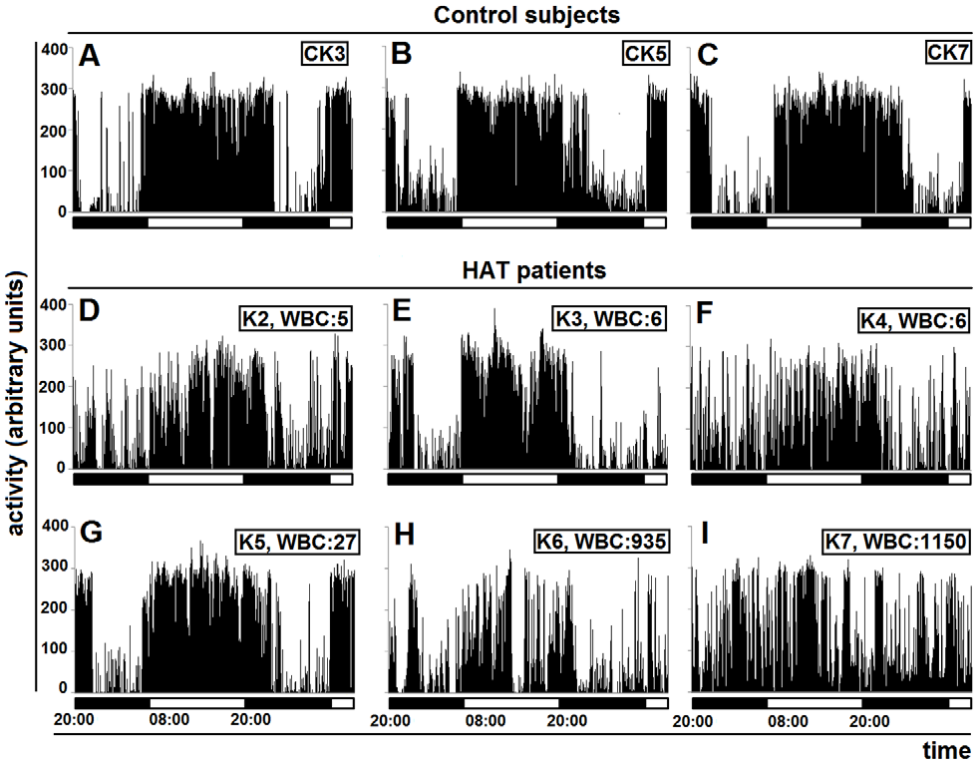
Actigrams of DRC adult patients before treatment. Actigrams are 36-h long and are those from the adult control subjects CK3, CK5, CK7 (A–C) and patients K2–K7 at admission (D–I). The horizontal bars indicate the light and dark periods. WBC: white blood cell counts in the cerebrospinal fluid.

**Figure 6:**
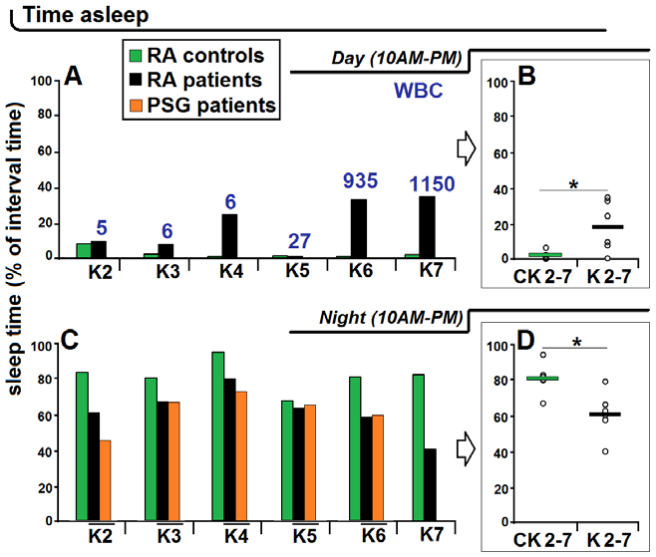
Total sleep time in the adult patients K2–K7. The analyses have been performed from actigraphic recordings (RA, rest-activity) during the day (A) and night (C) and compared with matched control subjects (CK2–CK7), and also from nocturnal polysomnography (PSG) (C). B and D show the statistical evaluation for the day and night values, respectively, and the horizontal bars indicate the mean value. As in Fig. 2, “day” corresponds to the suggestive daily time of maximal activity and “night” to the suggestive daily time interval of rest. The numbers refer to white blood cell (WBC) counts in the cerebrospinal fluid. In B and D: * P<0.05, Student's unpaired *t* test.

The actigrams of the adult patients compared to the matched controls showed varying degrees of alterations (Fig. 5). In the 2 patients with very high WBC number in the CSF (K6 and K7), the actigrams revealed a complete disruption of the day/night cycle (Fig. 5H,I). The analysis of sleep time showed that these patients had sleep episodes accounting for a considerable proportion of the day (K6: 31.8%, K7: 33.7% of sleep time *versus* less than 1% in the respective controls), and a considerable decrease of the time spent asleep during the night (K6: 57.2%, K7: 39.9%, *versus* about 80% in the respective controls) (Fig. 6A,C).

The alterations were less marked in most of the other adult patients (Figs. 5D–G, 6). However, when considering the severity of alterations revealed by actigraphy and the WBC counts in the CSF, discrepancies were noted. The actigrams appeared altered in 2 patients with WBC counts corresponding to 5 and 6 cells, respectively (Fig. 5D,F), while they were relatively preserved not only in another patient with a WBC count of 6 cells (Fig. 5E), but also in a patient with WBC count of 27 cells (Fig. 5G).

Quantitative analyses of the proportion of sleep time in the adult patients K2–K4 revealed corresponding alterations especially during the night (Fig. 6C), with a considerable increase of time spent asleep during the day in patient K4 (23.5% *versus* 0.6% in the control) (Fig. 6A). Analyses derived from simultaneous nocturnal PSG recordings in the adult patients K2–K6 showed a good correspondence with the RA signal in actigraphy, though the latter overestimated sleep time in one patient (K2) (Fig. 6C), as noted above for patient Y2.

The statistical analysis showed a significant difference in the average time spent asleep during day and night, respectively, in the group of adult HAT patients *versus* the control group, with significant increase of the total sleep time during the day and decrease during the night (P<0.05 for both parameters) (Fig. 6B,D).

**Figure 7:**
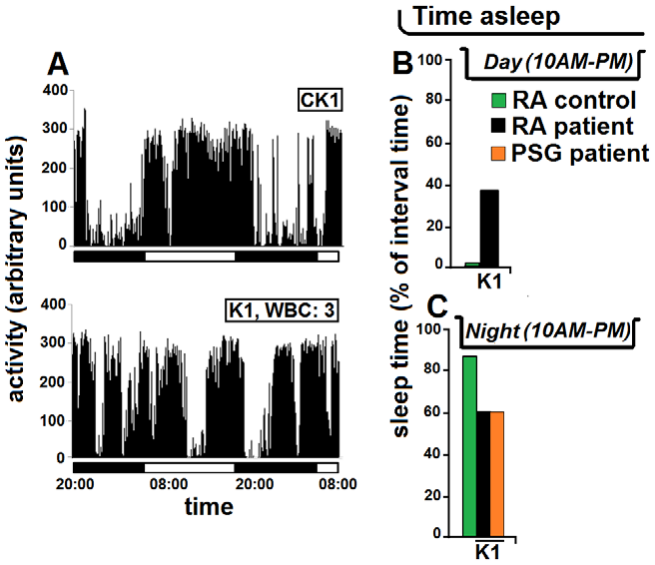
Actigram of a child before treatment. The actigrams, 36-h long, of a 5 year-old child affected by HAT (K1) and of a matched control child CK1 (A) together with evaluations of the total sleep time (B,C) are shown. The proportion of time spent asleep was evaluated during the day from actigraphic recording (RA, rest-activity) (B), and during the night from actigraphic and polysomnographic (PSG) recordings (C). The horizontal bars in A indicate the light and dark periods. WBC: white blood cell count in the cerebrospinal fluid.

In the 5-year old child infected by *T.b. gambiense* with a WBC count in the CSF of 3 cells, actigraphy revealed marked functional alterations compared with the healthy child of the same age and sex (Fig. 7). The actigram of this patient appeared disrupted (Fig. 7A), as reflected by the quantitative evaluation of the sleep time, which corresponded to 37.8% of the day (*versus* 0.76% in the control child) (Fig. 7B) and 62.7% of the night (*versus* 90% in the control) (Fig. 7C). The nocturnal PSG recording provided the same value of sleep time (62.4%) (Fig. 7C) as that derived from actigraphy.

SOREM episodes were observed in all DRC patients except in K6 (Fig. S2) (in K7, as mentioned previously, PSG data were corrupted and therefore not scored).

**Figure 8:**
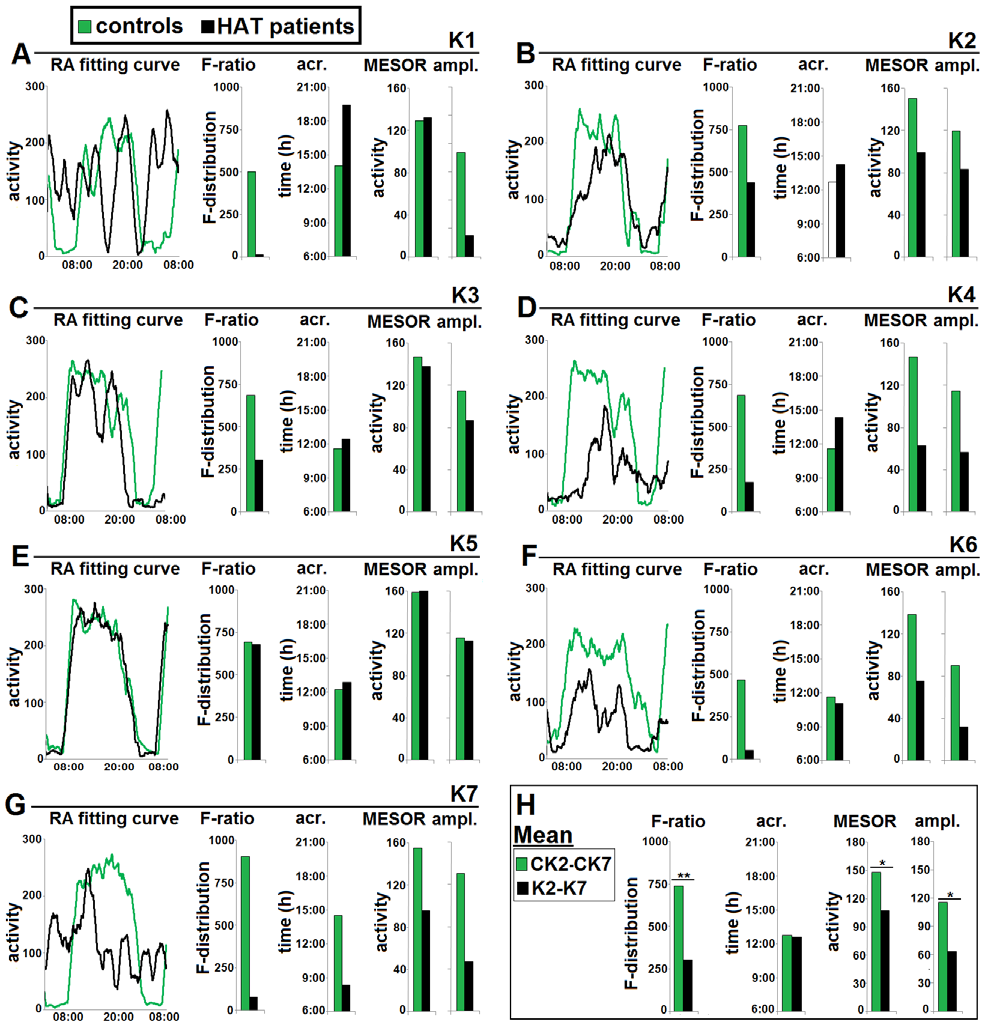
Characteristics of the rest-activity rhythm in DRC patients. The characteristics of the rest-activity (RA) rhythm in patients K1–K7 and control subjects (A–F) are shown together with the assessment of statistical difference between adult control subjects and patients (H). A–F. All the parameters have been evaluated as explained in the legend to Figure 4. Note the different degrees of alterations of the waveform (RA fitting curve), of the F-ratio, acrophase (acr.), MESOR, and amplitude (ampl.). **H**: the statistical evaluation shows significant intergroup differences of the F-ratio, acrophase, MESOR and amplitude; *P<0.05, **P<0.01, Student's unpaired *t* test. F*crit* = 3.68. For RA rhythms of all patients, F-ratio>F*crit*; P<0.000001.

The analysis of the rhythm waveform showed in the patients different degrees of alterations of the daily pattern and rhythm parameters (Fig. 8A–G). The statistical evaluation of the rhythm characteristics in the adult patients confirmed a significant mean decrease of the F-ratio with respect to the matched control group (P<0.01) (Fig. 8H). These analyses also showed a significant decrease of the mean values of the MESOR and amplitude (P<0.05 for both parameters), with a preserved mean value of acrophase time, thus indicating that the time of peak activity was less affected than the other rhythm characteristics (Fig. 8H).

## Discussion

Our pilot study shows that actigraphy during HAT can effectively reveal sleep-wake alterations characteristic of this disease and encourages further investigation. In particular, sleep-wake parameters, such as the night sleep disruption and the infiltration of episodes of sleep during the day, pointed out by previous PSG studies [15], were well reflected by the actigrams. Furthermore, simultaneous actigraphic and PSG recordings during the night have shown in the present study a good correspondence of the two techniques in revealing sleep disturbances.

The use of PSG has contributed to significant progress in the diagnosis of neurophysiological alterations during HAT [14]–[16], [20], and this technology remains the gold standard for the analyses of sleep-wake disorders. However, PSG may have serious limitations in HAT endemic settings. Compared to PSG, actigraphy requires much simpler and cost-effective equipment (a dry-cell battery-run, a watch-size device and a laptop). The software (5.5 megabytes) can run on a portable computer, and the files of the actigrams (21 kilobytes) can be easily transferred through a cellular phone, or attached to e-mail messages even with slow internet connections, making very easy the acquisition and transfer of data for analyses. The battery-run actigraph can record and store data for several days without the risk of data loss due to electrical power failures (which can frequently occur in field studies and did occur during our study). The possibility of an easy use of actigraphy in the field needs requires confirmation in future studies, but it should also be considered that can be done at home, and is thus well suited for long-term studies without interfering with the subject's routine activities [22].

In our investigation, the qualitative observation of actigraphic recordings showed in the cohort of 8 adult patients (Y1 and Y2, and K2–K7) varying degree of alterations. These ranged from well evident, high bouts of activity during the night, and therefore with considerable sleep fragmentation, to the complete disruption of the sleep-wake cycle. In patients Y1 and Y2, actigraphy showed an improvement of the signal parameters after treatment initiation. These findings tally with previous studies based on PSG recordings [20], which have in addition shown that complete recovery of sleep-wake parameters in HAT patients requires a very long time, and symptoms may persist for months after the end of treatment. A longitudinal long-term assessment is therefore required, actigraphy is especially suited for this purpose.

The present observation in the child affected by HAT (K1) is in accordance with previous evidence that actigraphy is valid for an evaluation of sleep-wake in children [29], [36]. This finding is also in accordance with a recent report that sleep alterations reflect HAT severity in children [19]. The present preliminary evidence of the efficacy of actigraphy in a child affected by HAT is of particular interest especially considering the great difficulty in the evaluation of clinical signs (such as inactivity and unusual behavior) in children of 1–6 years of age suffering from this disease [17].

Although actigraphy is not a routine procedure in the evaluation of brain and/or systemic infections, data have been obtained with actigraphic recording in persons living with HIV infection [39], [40], given that sleep disturbance is a common complaint during this infection. These studies have been performed in HIV-infected women with CD4 cell counts between 40 and 930 mm^3^, and using as exclusion criteria AIDS-dementia diagnosis, neuropathy or use of illicit drugs. The investigations have shown a moderate reduction of sleep time during the night with napping episodes during the day, providing an objective evaluation of the patients' complaints of insomnia and fatigue [39], [40]. These alterations do not configurate, however, the fragmentation of the sleep pattern and sleep-wake cycle characteristic of HAT.

Limitations of actigraphy were also evident in our pilot study. SOREM episodes, detected in the hypnograms of our HAT patients in agreement with previous findings [15], could not be differentiated from normal rest in the actigraphic recording. Furthermore, the simultaneous actigraphic and nocturnal PSG recordings showed that in some of the adult cases the analysis derived from actigraphy tended to overestimate the total sleep time. As mentioned above, this limitation is inherent to the technique, due to the difficulty to distinguish sleep from episodes in which the subject wakes up but remains motionless [22]. However, this limitation was also present in the evaluation of sleep from actigraphy in control subjects and in multiple sessions of actigraphic recordings, and may not, therefore, influence the patients' clinical monitoring. It should also be noted that in the child affected by HAT the sleep time derived from actigraphy and nocturnal PSG showed the same values, probably due to the fact that quiet wakefulness events are unlikely to occur in children, thus further supporting the use of this tool for the clinical evaluation of children affected by HAT.

An interesting issue is raised by the correlation between the signs of disease revealed by actigraphy and PSG on one hand, and the WBC counts in the CSF on the other hand. In agreement with previous investigation based on PSG [15], [16] the patients with very high number of WBCs in the CSF showed severe sleep-wake changes (here revealed by both actigraphy and PSG), while in the patients with lower numbers of WBCs (3–27 WBC/µl CSF in our study) such correlation was not close. In addition, it has to be noted that SOREM episodes were detected in our study also in patients with 3–6 WBC/µl CSF, as shown in a previous study in which SOREM episodes were detected in 2 out of 4 patients with 0–7 WBC/µl CSF, although the episodes were more frequent in patients with >400 WBC/µl CSF [15].

Importantly, HAT brings about a severe neuroinflammatory condition. Cytokines and chemokines are at the core of HAT pathogenetic and clinical parameters, and inflammatory mediators have been implicated in sleep disturbances during the disease [18], [41], [42]. Novel experimental evidence has pointed out potential laboratory biomarkers of HAT severity and stages [7], [9]–[13]. The correlation of actigraphic recordings and cytokine measurements has already been validated in other conditions which lead to increase of inflammatory biomarkers [43], [44], also in children [45]. Such correlation could thus represent a precious diagnostic and monitoring approach in HAT management.

On the basis of the results here presented, and despite the above limitations, actigraphy appears as a tool well suited for objective measurements of disturbed sleep pattern and the daily distribution of sleep-wake in HAT patients, providing valuable neurophysiological information. Considering the current interest to establish scoring scales adapted to the clinical characteristics of the various types of nervous system diseases, it is foreseen that such scoring scales will be developed in the near future also for HAT to improve the assessment of new treatments. Objective data obtained by user-friendly actigraphy, standardized in a larger cohort of HAT patients, could therefore be suited to be incorporated into such novel scoring systems.

In conclusion, on the basis of this first published report, actigraphy seems to be a very promising tool for obtaining objective clinical data in HAT, suited also for long-term assessment and follow-up of HAT patients. This technique could therefore also be useful for the clinical evaluation of relapses, assessment of novel treatments and correlation with disease and staging biomarkers in body fluids which are currently under investigation in many laboratories. This is of special relevance given that HAT affects populations living in environments with precarious health facilities.

## Supporting Information

Figure S1 **Raw data of actigrams and steps followed in data analysis.**
(TIF)

Figure S2 **Nocturnal hypnograms of patients K1–K7.**
(TIF)

Text S1 **Analyses of the rest-activity rhythm.**
(DOC)
