# Supplementary figures and images for: Actigraphy in Human African Trypanosomiasis as a Tool for Objective Clinical Evaluation and Monitoring: A Pilot Study
Source: PLoS Negl Trop Dis. 2012 Feb 14;6(2):e1525. doi: 10.1371/journal.pntd.0001525 (PMC3279345; doi:10.1371/journal.pntd.0001525)

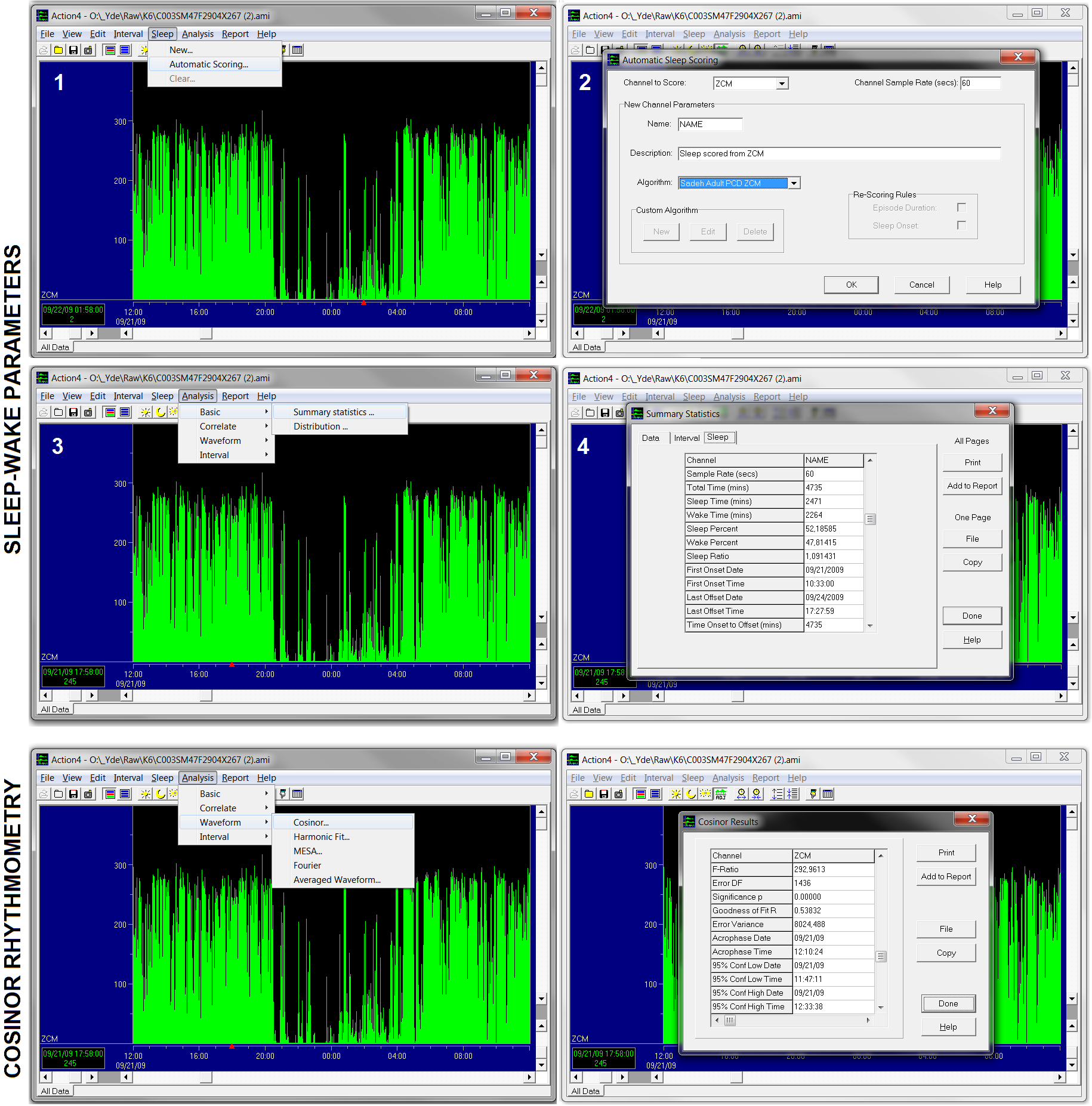

Supplement: Figure S1 — Raw data of actigrams and steps followed in data analysis. (TIF) [file pntd.0001525.s001.tif]

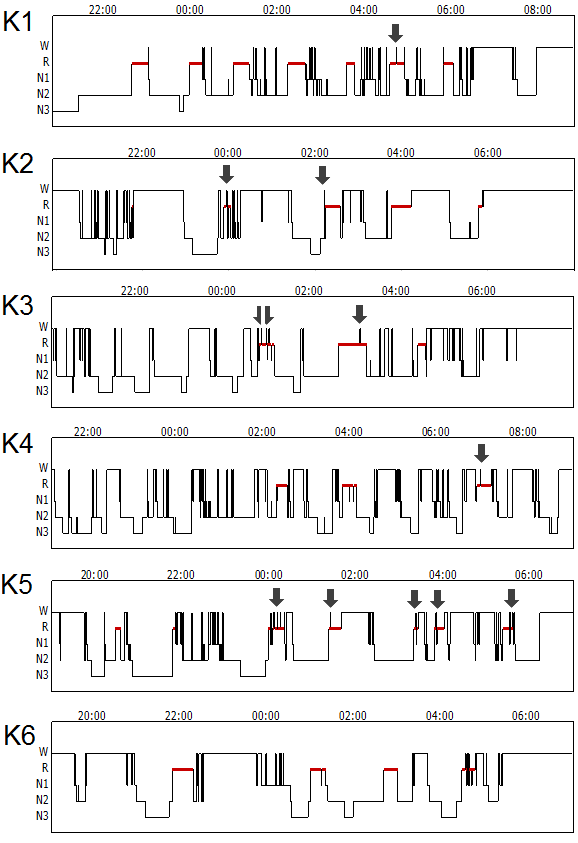

Supplement: Figure S2 — Nocturnal hypnograms of patients K1–K7. (TIF) [file pntd.0001525.s002.tif]
