# Supplementary material for: Actigraphy in Human African Trypanosomiasis as a Tool for Objective Clinical Evaluation and Monitoring: A Pilot Study
Source: PLoS Negl Trop Dis. 2012 Feb 14;6(2):e1525. doi: 10.1371/journal.pntd.0001525 (PMC3279345; doi:10.1371/journal.pntd.0001525)
Supplement: Text S1 — Analyses of the rest-activity rhythm. (DOC) [file pntd.0001525.s003.doc]

Supplementary Information S2:

**Analysis of the rest-activity rhythm**

The cosinor rhythmometry method was here used to study the rest-activity (RA) 24 h rhythm. This method allows the evaluation of the rhythm characteristics of the RA signal by determining the sinusoidal composite waveform that fits at best to the signal; this is graphically visualized by a curve which provides at a glance information on the patient’s rhythm characteristics (as shown in the “RA fitting curve” in Figs. 4 and 8). To determine the best fitting composite waveform, the cosinor rhythmometry method evaluates different possible composite waveforms comparing them to the RA signal with an F-test. The value rendered by the F-test is the F-ratio, which corresponds to the ratio of two variances arising from analysis of variance. The F-ratio value indicates the degree of fragmentation of the RA signal: the more the tracing is fragmented, the lower is the F-ratio associated. Only the composite waveform with the largest F-ratio is considered and processed, as that waveform is the closest to the real rhythm. The characteristics of the RA signal rhythm considered correspond therefore to the trigonometric characteristics of the composite waveform: midline estimating statistic of rhythm (MESOR), amplitude, acrophase (Halberget al. 2001;Nelson et al. 1979). The MESOR is the rhythm-adjusted mean (mean of the fitted curve); the amplitude is the difference between the maximum and the MESOR of the RA signal fitted curve (peak-to-nadir difference); the acrophase (time of peak activity) is the lag from a defined reference point (midnight in the present study) (Ancoli-Israel et al. 2003; Nelson, Tong et al., 1979). In the present analyses, the best fitting and significant sinusoidal composite waveform was determined automatically from the RA signal using the Action 4 software (see Fig. S1); the F-ratio and the associated rhythm characteristics were also obtained with this software.

**S2 References**

Ancoli-Israel S, Cole R., Alessi C., Chambers M., Moorcroft W et al. (2003) The role of actigraphy in the study of sleep and circadian rhythms. Sleep 26: 342-392.

Halberg FE, Cornelissen G, Otsuka K, Schwartzkopff O, Halberg J et al. (2001) Chronomics. Biomed.Pharmacother. 55 Suppl 1: 153s-190s.

Nelson W, Tong YL, Lee JK, Halberg F (1979) Methods for cosinor-rhythmometry. Chronobiologia 6: 305-323.
